# Real-time assessment of tissue oxygen saturation during endovascular therapy for chronic limb threatening ischemia using a novel oximeter

**DOI:** 10.1016/j.jvscit.2021.07.001

**Published:** 2021-07-22

**Authors:** Naoki Unno, Kazunori Inuzuka, Masaki Sano, Masatsugu Niwayama, Ena Naruse, Hiroya Takeuchi

**Affiliations:** a Division of Vascular Surgery, Hamamatsu University School of Medicine, Hamamatsu, Japan; b Second Department of Surgery, Hamamatsu University School of Medicine, Hamamatsu, Japan; c Division of Vascular Surgery, Hamamatsu Medical Center, Hamamatsu, Japan; d Department of Electrical and Electronic Engineering, Shizuoka University, Hamamatsu, Japan

**Keywords:** Chronic limb-threatening ischemia, Endovascular treatment, Foot ulcer, Near-infrared spectroscopic oximeter, Regional oxygen saturation monitoring

## Abstract

In the present study, we have introduced a novel real-time, near-infrared spectroscopy oximeter, the TOE-20 (Astem, Co, Ltd, Kawasaki, Japan), which can simultaneously measure the regional tissue oxygen saturation (rSO_2_) in the skin and subcutaneous tissue at three angiosomes of the foot. Seven patients with chronic limb threatening ischemia who had undergone successful revascularization of the superficial femoral artery were included. The analysis revealed a significant correlation between the rSO_2_ and skin perfusion pressure. After revascularization, the rSO_2_ and skin perfusion pressure had both increased at the three regions, although the increase at the plantar foot was insignificant. These results indicate that the TOE-20 can be successfully used to monitor the rSO_2_ during endovascular treatment.

Owing to the increasing incidence of diabetes and renal insufficiency, the number of patients with chronic limb threatening ischemia (CLTI) has increased to >6 million globally.^1^ Endovascular therapy (EVT) is a major treatment of CLTI, especially for patients with a high surgical risk.^2^ Previously, we introduced the use of a finger-mounted tissue oximeter that relies on near-infrared spectroscopy (NIRS) techniques (Toccare; Astem Co, Ltd, Kawasaki, Japan) to assess ischemia severity in patients with peripheral artery disease.^3^ Subsequently, the team developed an NIRS device to simultaneously facilitate intra-EVT monitoring of tissue oxygenation at multiple sites. In the present report, we have described this novel device and presented our preliminary results for the real-time monitoring of regional tissue oxygen saturation (rSO_2_) in the skin and subcutaneous tissue at three angiosomes of the foot during EVT.

## Methods

### Study approval

The ethical committee of Hamamatsu University School of Medicine approved the present study (approval no. 16-057). The study protocol was registered at the UMIN (university hospital medical information network) clinical trials registry (identification no. UMIN000025021) and Japan registry of clinical trials (identification no. CRB4180008). All the participants provided written informed consent.

### Tissue oximeter

**Supplementary Fig 1:**
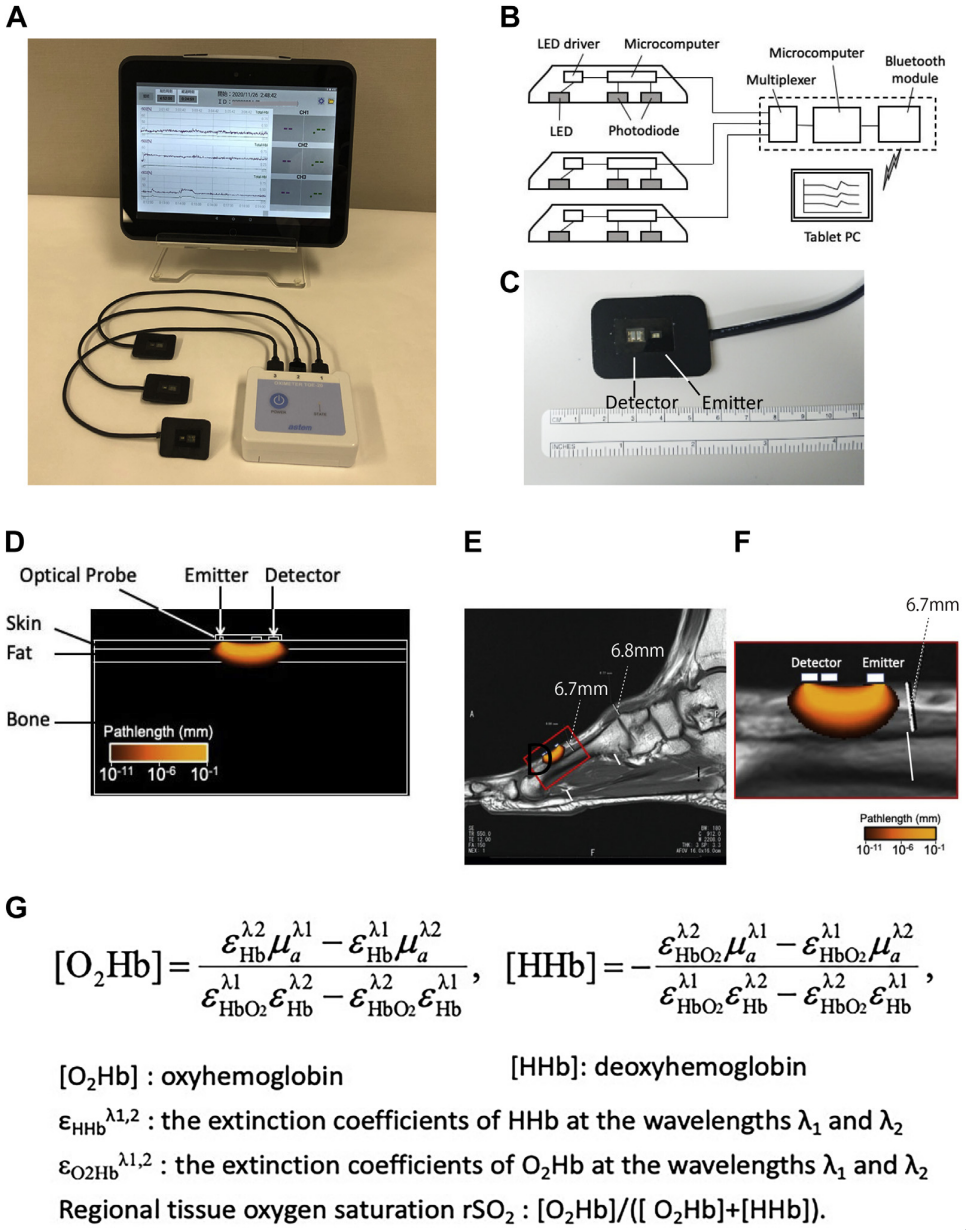
Novel tissue oximeter TOE-20 (“target region oxygenation-based endovascular treatment”; Astem, Co, Ltd, Kawasaki, Japan). **A,** Photograph of the TOE-20 oximeter. **B,** Engineering schematic for the TOE-20. **C,** Sensor probe of the TOE-20. **D,** Model for the Monte Carlo analysis and sensitivity distribution obtained via simulation. **E,** Path length distribution superimposed on a typical magnetic resonance image of the foot. **F,** Magnified image of the area surrounded by the *red-colored square* in E. The depth of the bone from the skin surface was 6.7 mm at the dorsum of the foot. **G,** The equations used to calculate the concentrations of oxyhemoglobin (*O*_*2*_*Hb*) and deoxyhemoglobin (*HHb*).

The new NIRS oximeter (TOE-20; Astem, Co, Ltd) enables real-time monitoring of the rSO_2_ (Supplementary Fig 1, *A*). The device's name, TOE, is an abbreviation for “target region oxygenation-based endovascular treatment,” which was previously proposed as a new strategy for EVT.^4^ The oximeter comprises three components: a small, box-shaped body (70 × 72 × 25 mm; weight, 120 g) that includes a multiplexer, microcomputer, blue-tooth module, and two AA batteries; three sensor probes with 40-cm long cables; and a tablet personal computer that displays the measurement results (Supplementary Fig 1, *B*). Each probe has near-infrared light-emitting diodes (770 nm and 830 nm) and detectors (photodiodes; Supplementary Fig 1, *C*). The path length distribution obtained from the simulation results is shown in Supplementary Fig 1, *D*. The path length distribution superimposed on a typical magnetic resonance image of the foot is shown in Supplementary Fig 1, *E* and *F*. The equations used to calculate the concentrations of oxyhemoglobin and deoxyhemoglobin are shown in Supplementary Fig 1, *G*.

### Application to patients

**Supplementary Fig 2:**
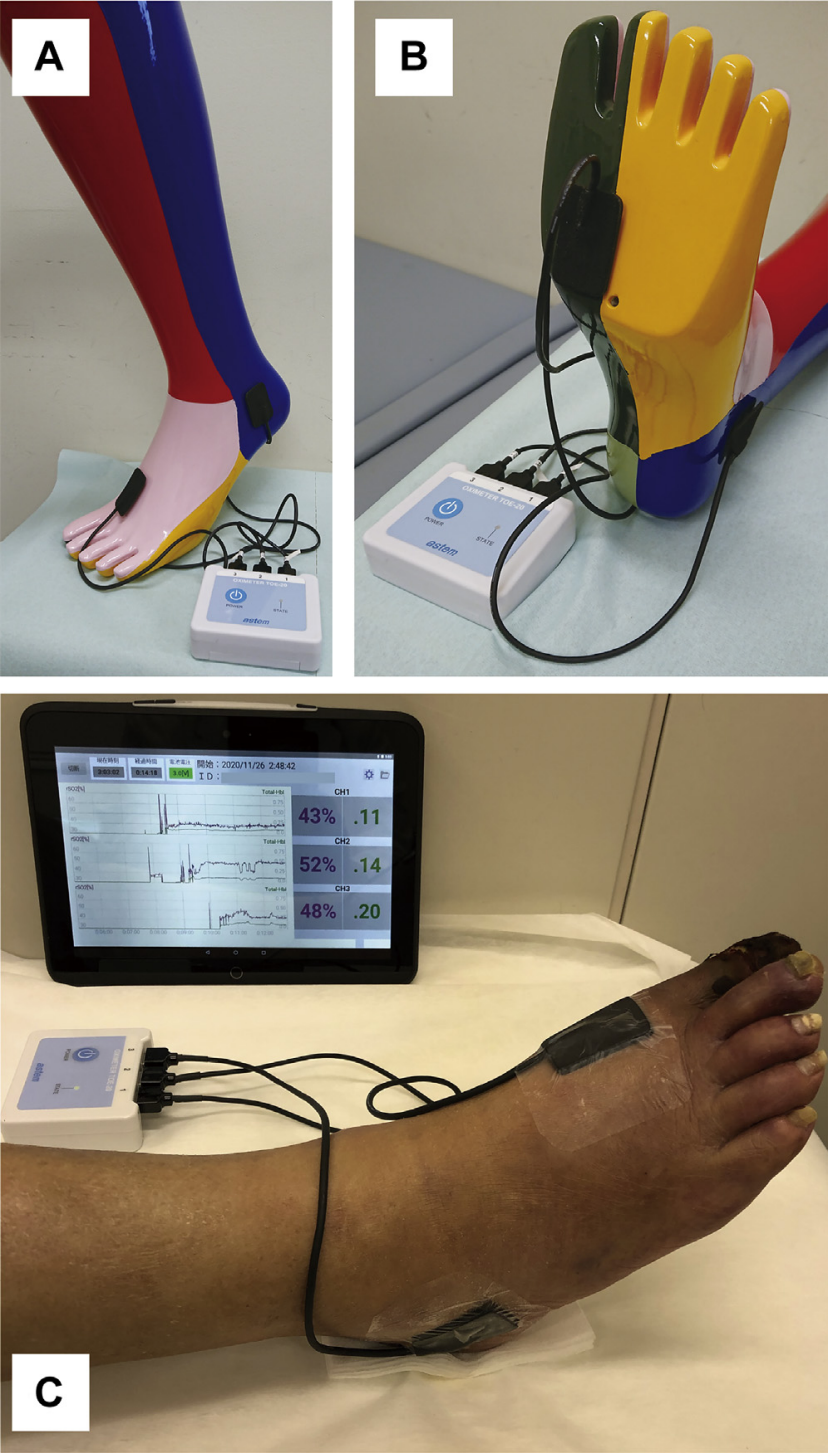
TOE-20 (“Target region Oxygenation-based Endovascular treatment”; Astem, Co, Ltd, Kawasaki, Japan) application to patients. **A,** Two sensor probes of the TOE-20 are attached to the dorsum of the foot and outer ankle of the angiosome model. **B,** Two sensor probes of the TOE-20 are attached to the plantar foot and outer ankle of the angiosome model. **C,** Simultaneous measurement of regional tissue oxygen saturation (rSO_2_) at the three angiosome sites of the foot using the TOE-20 in a patient with chronic limb threatening ischemia (CLTI).

Three sensor probes can be placed at the operator's discretion to monitor tissue perfusion. The placement of probes according to the angiosome model is shown in Supplementary Fig 2: one on the dorsal aspect of the foot, one on the outer ankle (Supplementary Fig 2, *A*) and one on the plantar aspect of the foot (Supplementary Fig 2, *B*). The actual monitoring setup for measuring rSO_2_ during EVT is shown in Supplementary Fig 2 *C*.

### Participants

The present prospective study included seven patients with CLTI who had undergone successful revascularization of the superficial femoral artery (SFA) without intervention in the tibial arteries and five patients with CLTI who had undergone failed EVT (four with failed tibial artery intervention and one with failed SFA intervention; Table). All the patients were categorized as having Rutherford classification 5 with intractable toe ulcers. Successful revascularization of the SFA was defined as <30% residual stenosis of the target lesion on the completion angiogram without peripheral emboli. The rSO_2_ values were measured within 0.5 second per point. Although the rSO_2_ was monitored continuously, we waited for 5 minutes to observe the effect of revascularization after each procedure because the values required a few minutes to stabilize (Fig, *A*). The skin perfusion pressure (SPP) was also measured at the same regions in the ambulatory clinic before and after treatment using an SPP system (SensiLase PAD 3000; Vasamed, Eden Prairie, Minn).

**Table:** Demographic and clinical characteristics

Characteristic	EVT	
	Successful	Failed
Patients	7	5
Age, years	76 (62-85)	74 (57-85)
Male sex	4 (57)	3 (60)
Hypertension	5 (71)	6 (60)
Dyslipidemia	3 (43)	6 (60)
Diabetes mellitus	5 (71)	6 (60)
History of smoking	5 (71)	8 (80)
End-stage renal disease	4 (57)	6 (60)
Coronary artery disease	2 (29)	6 (60)
Limbs treated	7	5
Ulcer location		
Toe	7	4
Plantar foot	0	1
Outer ankle	0	0
Ankle brachial index	0.63 ± 0.13	0.58 ± 0.25

*EVT,* Endovascular treatment.

Data presented as number, median (interquartile range), number (%), or mean ± standard deviation.

**Fig:**
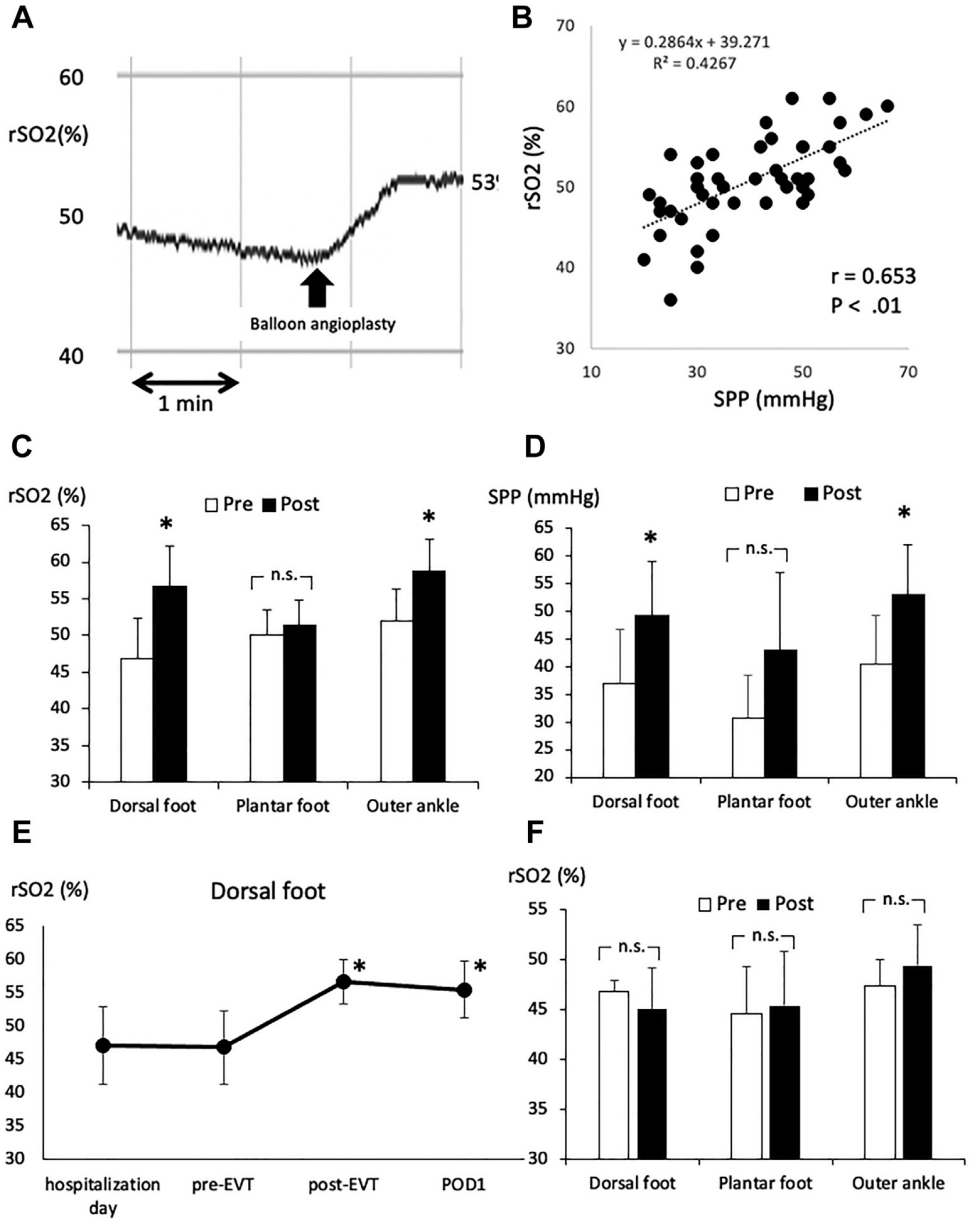
Regional tissue oxygen saturation (*rSO*_*2*_). **A,** The rSO_2_ curve for one patient during the endovascular procedure. The rSO_2_ values had stabilized after a few minutes. **B,** Scatter plot showing the relationship between the rSO_2_ and skin perfusion pressure (*SPP*) at the surface of the foot (*P* < .01; *r* = 0.653). The values reflect the data from all seven patients who had undergone successful superficial femoral artery (SFA) revascularization via endovascular treatment (EVT). **C,** Comparison of rSO_2_ values at the dorsal foot, plantar foot, and outer ankle before and after EVT in seven patients. ∗*P* < .01 compared with before EVT. **D,** Comparison of SPP values at the dorsal foot, plantar foot, and outer ankle before and after EVT in seven patients. ∗*P* < .01 compared with before EVT. **E,** Changes in rSO_2_ values at the dorsal foot in seven patients who had undergone successful SFA EVT on the day of hospitalization and before EVT, after EVT, and on postoperative day 1. ∗*P* < .05 compared with before EVT. **F,** Comparison of rSO_2_ values at the dorsal foot, plantar foot, and outer ankle before and after EVT in five patients who had undergone failed EVT. *n.s.,* Not significant.

### Statistical analysis

The correlations between the rSO_2_ and SPP values were analyzed using the nonparametric Spearman rank correlation tests. The results are presented as the mean ± standard deviation. Paired *t* tests were used to compare the rSO_2_ and SPP values from before and after revascularization at the same sites. One-way analysis of variance and nonparametric Friedman tests with the post hoc Tukey test were used to examine differences in the time course of the rSO_2_ values in the dorsal foot among the seven patients. The level of statistical significance was set at *P* < .05 (SPSS, version 25.0 software; IBM Corp, Armonk, NY).

## Results

SFA revascularization was successfully performed via endovascular intervention with balloon angioplasty in five patients, a Viabahn stent-graft (W.L. Gore & Associates, Flagstaff, Ariz) in one patient, and stent placement in one patient. In the outflow arteries below the knee, completion angiography revealed two run-off vessels in four patients and one run-off vessel in three patients. Therefore, all seven patients had at least one straight-line flow found on the below-the-knee angiogram. After revascularization, the sensor probes gradually responded to the increased blood flow to increase the rSO_2_ to a stable value. A significant correlation was found between the SPP and rSO_2_ values (*P* < .01; Fig, *B*). After revascularization, the rSO_2_ and SPP had both increased at the dorsal foot and outer ankle (Fig, *C* and *D*). The time course of the rSO_2_ values in the seven patients on the day of hospitalization, before EVT, after EVT, and on postoperative day 1 are shown in Fig. *E*. By 3 months after EVT, all the ulcers had healed in all seven patients. In contrast, no significant increase was found in the rSO_2_ at any region in the patients with failed EVT (Fig, *F*).

## Discussion

The present results have demonstrated the utility of the TOE-20 for simultaneous monitoring of the rSO_2_ in the skin and subcutaneous tissue in three angiosomes of the foot during EVT. Previous NIRS oximeters focused on measuring oxygen levels in the brain or muscles at a depth of 10 to 20 mm below the skin surface.^5^ However, these devices might not be able to measure the oxygen levels in the skin and subcutaneous tissue of the toe because bones or tendons are present at depths <10 mm from the skin surface. In contrast, the TOE-20 is specifically designed to measure the rSO_2_ in the skin and subcutaneous tissue. The superimposed magnetic resonance image of the foot revealed that the TOE-20 reflects the rSO_2_ levels to ≤5 mm under the skin surface and can measure the rSO_2_ of the skin and subcutaneous tissue without the influence of bone. This characteristic is quite unique compared with the other NIRS devices, which mainly measure the cerebral oxygen levels using an algorithm that diminishes the contribution of the skin and scalp.^6^ One of the greatest advantages of the TOE-20 is the speed with which the rSO_2_ can be measured. Continuous monitoring allows one to obtain the values almost instantly (within ∼0.5 second), reducing the influence of the patient's bodily movements on the measurements. High and low rSO_2_ areas were observed even within the same angiosome, because peripheral tissue perfusion in the foot of patients with CLTI (especially those with diabetes) is determined by the peripheral microvascular blood flow in the skin and subcutaneous tissue.^7^ After revascularization, the increases in the rSO_2_ and SPP at the plantar foot were not significant. The lack of a patent pedal arch in some of the patients might explain why the plantar rSO_2_ did not increase significantly after EVT. Because all seven patients had toe ulcers, we had assumed that the rSO_2_ in the dorsal foot most likely reflected the oxygenation in the toe ulcers.

We previously investigated the use of a finger-mounted oximeter that relies on the same algorithm as the TOE-20 in 34 patients with CLTI without infection who had undergone EVT. All patients with an rSO_2_ of ≥50% in the dorsal foot on postoperative day 1 exhibited improved ulcer healing, indicating that an rSO_2_ of ≥50% might be a cutoff value for wound healing.^8^ Further studies with longer observation periods are required to verify the cutoff value of rSO_2_ for wound healing.

## References

[1] Biancari F. (2013). Meta-analysis of the prevalence, incidence and natural history of critical limb ischemia. J Cardiovasc Surg.

[2] Aboyans V., Ricco J.B., Bartelink M.E.L., Björck M., Brodmann M., Cohnert T. (2018). Editor’s choice – 2017 ESC guidelines on the diagnosis and treatment of peripheral arterial diseases, in collaboration with the European Society for Vascular Surgery (ESVS). Eur J Vasc Endovasc Surg.

[3] Yata T., Sano M., Kayama T., Naruse E., Yamamoto N., Inuzuka K. (2019). Utility of a finger-mounted tissue oximeter with near-infrared spectroscopy to evaluate limb ischemia in patients with peripheral arterial disease. Ann Vasc Dis.

[4] Unno N., Inuzuka K., Sano M., Kayama T., Naruse E., Takeuchi H. (2020). Target region oxygenation-based endovascular treatment in a chronic limb-threatening ischemia patient with multifocal arterial diseases. J Vasc Surg Cases Innov Tech.

[5] Rao R., Saint-Cyr M., Ma A.M., Bowling M., Hatef D.A., Andrews V. (2009). Prediction of post-operative necrosis after mastectomy: a pilot study utilizing optical diffusion imaging spectroscopy. World J Surg Oncol.

[6] Hyttel-Sorensen S., Sorensen L.C., Riera J., Greisen G. (2011). Tissue oximetry: a comparison of mean values of regional tissue saturation, reproducibility and dynamic range of four NIRS-instruments on the human forearm. Biomed Opt Express.

[7] Kagaya Y., Ohura N., Suga H., Eto H., Takushima A., Harii K. (2014). “Real angiosome” assessment from peripheral tissue perfusion using tissue oxygen saturation foot-mapping in patients with critical limb ischemia. Eur J Vasc Endovasc Surg.

[8] Kayama T., Sano M., Yata T., Inuzuka K., Katahashi K., Yata T. (2021). A pilot study investigating the use of regional oxygen saturation as a predictor of ischemic wound healing outcome after endovascular treatment in patients with chronic limb-threatening ischemia. Ann Vasc Dis.

